# Effect of vision training on visual function in older adults: A pilot randomized trial

**DOI:** 10.1371/journal.pone.0356206

**Published:** 2026-08-24

**Authors:** Daisuke Sudo, Daisuke Toyoda

**Affiliations:** 1 National Institute of Advanced Industrial Science and Technology (AIST), c/o Kashiwa II Campus, University of Tokyo, Kashiwa, Japan; 2 Department of Physical Therapy, School of Health Sciences at Odawara, Interna-tional University of Health and Welfare, Odawara-shi, Kanagawa, Japan; Aichi Prefectural Mikawa Aoitori Medical and Rehabilitation Center for Developmental Disabilities, JAPAN

## Abstract

Falls among older adults are a major public health concern; however, scientific evidence for interventions that specifically target visual function remains limited. Therefore, this exploratory pilot randomized trial evaluated changes in multiple visual-function outcomes associated with an oculomotor-focused vision training (VT) program in community-dwelling older adults. Thirty community-dwelling older adults (mean age, 73.7 ± 4.8 years) were randomly assigned to a lower-limb strength training group (SLR) or a group with SLR and an additional VT component (SLR + VT). Over 8 weeks, six visual-function components were assessed using the V-training system (eye–hand coordination, visual memory, spatial cognition, peripheral perception, central/peripheral perception, and eye movement). Physical function was assessed using the Timed Up and Go test. For each outcome, ANCOVA was performed with the post-intervention value as the dependent variable, group as the fixed factor, and the corresponding baseline value as a covariate. Baseline-adjusted analyses showed no clear between-group differences in post-intervention visual-function or physical-function outcomes. Several point estimates favored the VT group for outcomes aligned with repeated eye-movement practice, but confidence intervals were wide. In this pilot trial, clear baseline-adjusted differences between the two SLR-based programs, with or without an additional VT component, were not confirmed. Given the small sample size, findings are hypothesis-generating and will inform the design of adequately powered trials with time- and attention-matched control conditions and clinically meaningful endpoints.

## Introduction

Falls in older adults are a major cause of fatal injuries. Approximately 27.5% of older adults experience a fall within one year, and nearly half of these incidents lead to fractures [1]. In 2015, the U.S. healthcare system spent more than 50 billion dollars related to fall injuries [2]. Even among relatively independent older adults, the risk of falling increases with age. Early identification of high-risk individuals and timely implementation of preventive interventions are therefore essential [3]. Common indicators for predicting falls include lower-limb muscle strength (iliopsoas, quadriceps, triceps surae), fall history, single-leg standing time, and the Timed Up and Go (TUG) test. These measures are used to classify older adults into high-risk or low-risk groups. However, many older adults continue to fall despite receiving training programs designed to improve these parameters during fall-prevention interventions.

Falls among older adults occur in a wide range of daily activities, including walking, transfers, and turning movements. Among these situations, walking-related falls often involve contact with or tripping over environmental obstacles. Safe obstacle avoidance requires rapid postural and gait adjustments to meet environmental demands. Older adults at high risk of falling frequently exhibit reduced ability to modify movement strategies. Sudden changes in step length may occur before obstacle clearance, causing postural instability and elevating the likelihood of stumbling [4,5]. These adaptive movement strategies rely heavily on accurate sensory input to appropriately adjust gait and posture in response to environmental demands. Among these sensory inputs, visual information plays a critical role in the execution of daily activities [6–8].

Recently, vision training (VT) has been used to enhance athletic performance. VT is intended to improve visual skills, particularly in athletes, to support higher performance levels. Advanced visual processing ability and fast response speed are critical for superior performance and can be improved through VT regardless of competitive level [9]. VT requires repeated engagement of eye movements, visual attention, and visuospatial processing, and such demands have been suggested to facilitate activity in neural regions related to visual processing [10]. In addition, VT has been reported to improve the allocation and switching of visual attention across the visual field [11]. However, the effects of VT in older adults have not been fully examined. Existing work in this population has explored movement improvement through gaze control, postural control interventions using visual feedback, and sensor-based visual feedback [12–16]. These interventions primarily target gaze control or visual feedback, with limited focus on visual function itself, which is essential for everyday mobility.

Although VT has demonstrated benefits in sports [17], its effects on eye–hand coordination (EHC), visual memory, peripheral perception, and eye movement in older adults remain unclear. In studies involving older adults, interventions have predominantly emphasized lower-limb strength and balance training, whereas visual function has often been addressed indirectly or not at all. Training approaches that directly target visual and oculomotor functions involved in environmental perception and movement planning remain insufficiently studied, and scientific evidence in this area is limited. Therefore, this study explored whether VT might be associated with changes in visual-function outcomes in older adults.

## Materials and methods

### Participants

This study included 30 community-dwelling older adults with no impairments in visual function or walking ability (mean age, 73.7 ± 4.82 years). Inclusion criteria were: (1) ability to walk independently, (2) sufficient visual function to perform daily activities without difficulty, and (3) absence of physical or cognitive impairments.

Participants were recruited between January–March 2024. Baseline assessments and interventions occurred from April–August 2024. Each participant completed an 8-week intervention with post-intervention assessments conducted immediately afterward. The sample size (15 participants per group) was determined pragmatically based on the anticipated recruitment capacity and available resources. The study was designed as an exploratory pilot trial to inform future research [18,19].

### Blinding

Due to the nature of the intervention, participant blinding was not feasible. Outcome assessors and data analysts were not blinded to group allocation. Therefore, the possibility of performance and detection bias cannot be excluded.

### Experimental procedure

**Fig 1:**
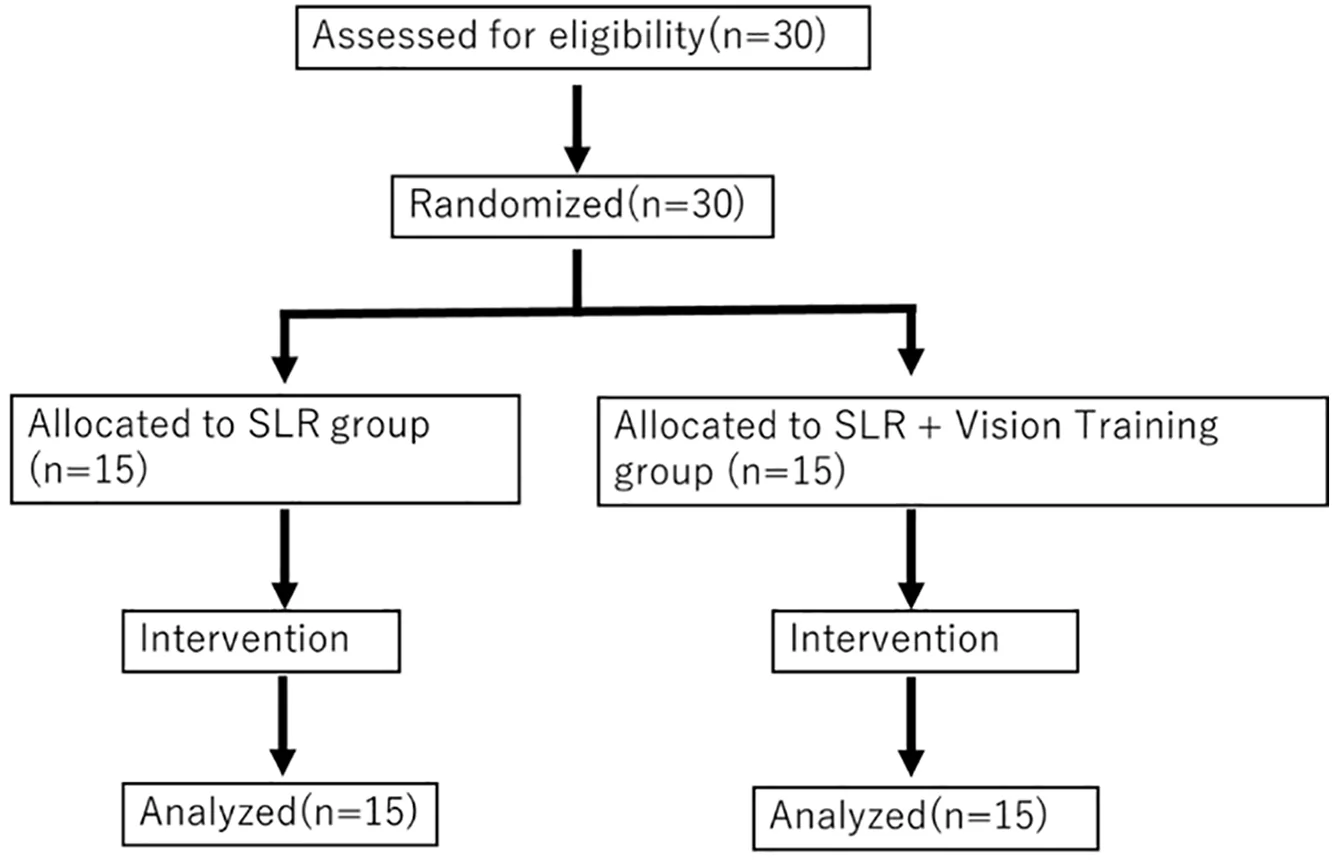
Flow diagram of participant enrollment, randomization, intervention, and analysis.

Thirty older adults were randomly assigned to two groups using a random number table (Fig 1). Simple randomization was performed without blocking or stratification. No allocation concealment mechanism (e.g., sealed envelopes or central randomization) was used. The investigator generated the random sequence and enrolled and assigned participants. Participants were allocated to the straight leg raising group (SLR group) or the combined SLR and VT group (SLR + VT group).

This study was approved by the Ethics Committee of International University of Health and Welfare (Approval No. 23-Ig-12) and registered in the UMIN Clinical Trials Registry (UMIN000050796). Written informed consent was obtained from all participants prior to enrollment in the study.

### Measures

**Fig 2:**
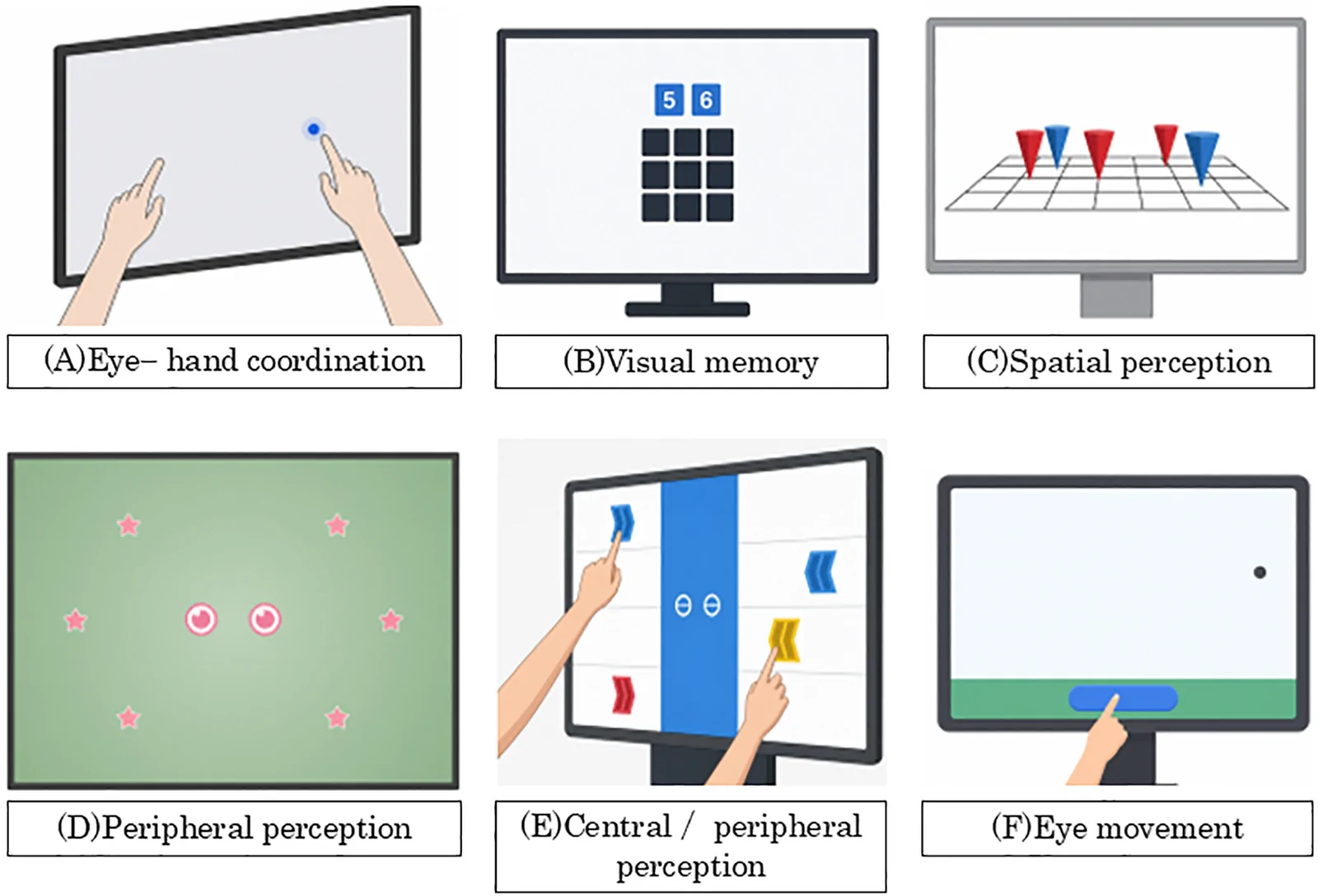
Visual function tasks assessed using V-training. (A) Eye–hand coordination: participants touch circular targets appearing at random as quickly and accurately as possible. (B) Visual memory: numbers are briefly presented and participants recall their positions. (C) Spatial perception: participants identify the final location of a moving target after it disappears. (D) Peripheral vision: participants detect faster rotating stars in the periphery while fixating centrally. (E) Central or peripheral perception: participants match the color of centrally presented stimuli with moving peripheral targets. (F) Eye movement: participants release their finger from the bar when the color of the moving target changes. Illustrations are author-created schematics for explanatory purposes.

Visual function was assessed using the V-training system (Advance Vision Partners LLC, Tokyo, Japan), which included six tasks: (1) eye–hand coordination, (2) visual memory, (3) spatial cognition, (4) peripheral perception, (5) central or peripheral perception, and (6) eye movement (Fig 2).

**Fig 3:**
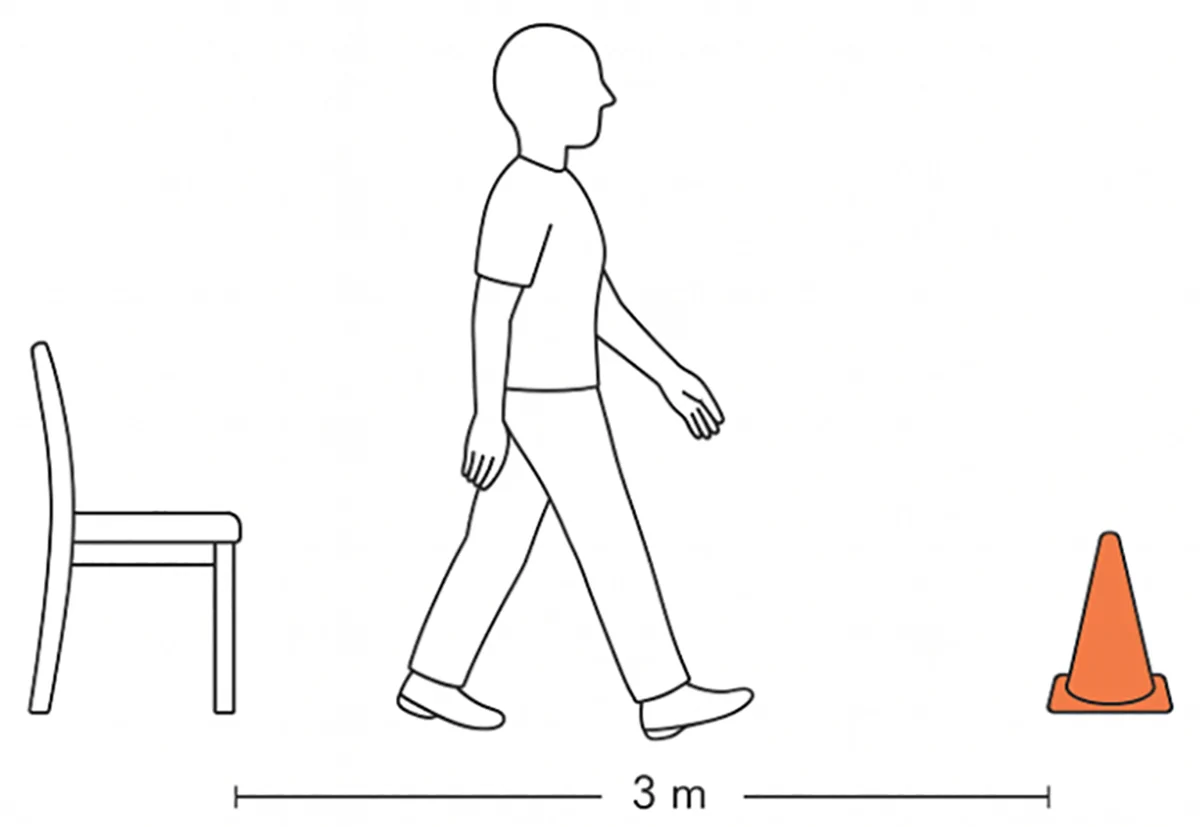
Measurement procedure for the Timed Up and Go (TUG) test. Participants stood up from a chair, walked 3 m to a cone, turned around, and sat back down.

Physical function was evaluated using the TUG test (Fig 3).

### Exploratory outcomes

Given the exploratory nature of this pilot trial, eye–hand coordination (EHC) was treated as a key exploratory visual-function outcome, as it reflects the efficiency of visual search and visuomotor response, which are plausibly influenced by repeated oculomotor eye-movement training. The remaining visual-function measures (visual memory, spatial cognition, peripheral perception, central/peripheral perception, and eye movement), as well as the Timed Up and Go test, were treated as additional exploratory outcomes.

### Visual function assessment with V-training

Visual function was evaluated using the V-training system in measurement mode.

### EHC

Participants stood approximately 40 cm from a 50-inch touchscreen monitor and visually tracked and touched 10 circular targets presented at random positions across central and peripheral visual fields. They were required to detect stimuli outside the direct line of gaze and respond rapidly. Participants eliminated all 10 targets as quickly as possible. The task was repeated three times, and the total completion time across the three trials was recorded as performance.

### Visual memory

Visual memory was assessed using a 3 × 3 grid displaying numbers 1–9 for 0.2 seconds at the center of the screen. The numbers subtended approximately 4 degrees of visual angle at a viewing distance of 1 m (image size = W117 px × H117 px). Immediately after presentation, participants recalled and reported two randomly selected numbers. The task was completed three times, and the highest accuracy score across trials was recorded.

### Spatial cognition

Spatial cognition was evaluated using a grid containing 4 vertical × 6 horizontal cells, within which a red conical target appeared randomly. After the target disappeared, the grid switched to a bird’s-eye view, and participants indicated the target location by touching the screen. The 3D target dimensions were X = 12.9 cm, Y = 16.3 cm, and Z = 12.9 cm. The task was repeated three times, and the highest accuracy score was recorded.

### Peripheral perception

Participants maintained fixation at the screen center. Six star-shaped targets appeared, three on each side, rotating as they radiated outward. One target on either side, or two targets (one on each side), shifted to a faster rotation speed. After three trials, participants identified and touched the fastest rotating target(s). The highest accuracy score across trials was recorded.

### Central/peripheral perception

Participants fixated both eyes on the center of the screen. From both edges, four colored targets (red, blue, yellow) appeared randomly and moved horizontally toward the blue central area, disappearing upon entry. A central color cue appeared irregularly, and participants touched the moving target(s) that matched the cue. Accuracy rate served as the measure. Targets were 2D images sized W105.6 px × H105.6 px.

### Eye movements

Participants placed a finger on a bar-shaped display at the bottom center of the screen. A target moved linearly in multiple directions, and participants tracked it visually. When the target color changed from black to white, participants rapidly released their finger and then returned it to the bar to continue.

Each set included three color changes, and three sets were completed. Reaction time for each trial and set was recorded to evaluate response speed during target movement. Targets were 2D images sized W15 px × H15 px.

### TUG

The TUG test used a 3-m walkway with a cone placed at the 3-m mark. At the start signal, participants stood up from a chair, walked to the cone, turned around, and returned to sit. Completion time was recorded [20]. Participants performed two trials, and the mean of the two trials was used for the analysis.

### Intervention

**Fig 4:**
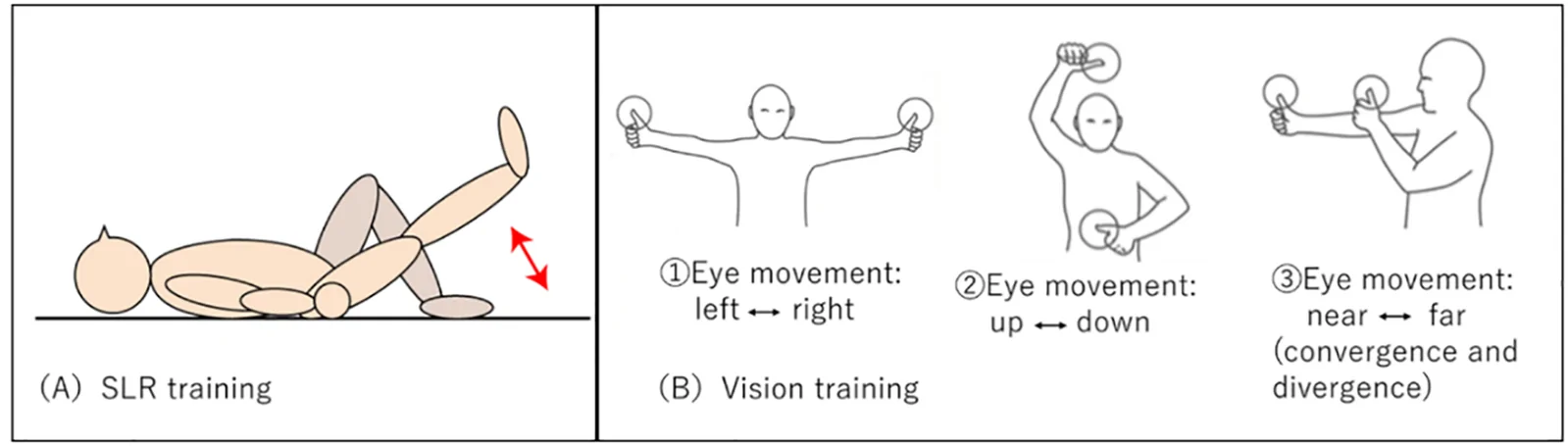
(A)SLR training (B)Vision training.

Participants performed either SLR or SLR + VT three times per week for 8 weeks(Fig 4A and 4B). After the intervention, the same six visual function tasks and physical function assessment were administered again. VT followed recommendations from the Japan Sports Vision Association and included three eye-movement exercises: (1) horizontal movements, (2) vertical movements, and (3) anteroposterior movements (Fig 4B). Participants held a fingertip 30 cm in front of the eyes and avoided forward head tilt. To prevent dizziness, unsteadiness, or falls, all exercises were completed in a seated position.

### Vision Training (VT) intervention

The VT program was conducted 3 times per week for 8 weeks (Fig 4B). Each session consisted of three oculomotor exercises: horizontal (left–right), vertical (up–down), and near–far eye movements (convergence and divergence). For each exercise, participants performed 10 back-and-forth repetitions × 2 sets. All training was conducted in a seated position under supervision to ensure safety. No progression of exercise intensity was implemented during the intervention period.

### Lower-limb strength training intervention

Lower-limb strength training in both groups consisted of straight leg raising exercises performed in the supine position (Fig 4A). Participants performed 2 sets of 10 repetitions for each leg (right and left) per session. Training sessions were conducted 3 times per week for 8 weeks. The exercise protocol was identical in both groups.

### Statistical analysis

For each outcome, analysis of covariance (ANCOVA) was used with the post-intervention value as the dependent variable, group (SLR vs. SLR + VT) as the fixed factor, and the corresponding baseline value as a covariate to estimate baseline-adjusted between-group differences. Adjusted mean differences with 95% confidence intervals are presented to describe the direction and uncertainty of group differences. Analyses were performed using SPSS Statistics version 27.0 (IBM Corp., Armonk, NY, USA).

## Results

All participants completed follow-up, and none were excluded after randomization (Fig 1). Participant characteristics are summarized in Table 1.

**Table 1:** Baseline characteristics of participants.

Variable	SLR group (n = 15)	SLR + VT group (n = 15)
Age, years (mean ± SD)	73.5 ± 4.67	73.9 ± 5.29
Sex, female/male	14/1	13/2
Visual acuity (self-reported)	All participants reported normal daily vision	
TUG, s (mean ± SD)	6.97 ± 0.89	6.90 ± 0.70

Baseline-adjusted comparisons using ANCOVA showed no clear between-group differences in post-intervention outcomes (Table 2).

**Table 2:** ANCOVA results for visual-function and physical-function outcomes.

Outcome (post)	Adjusted mean (SE) SLR	Adjusted mean (SE) SLR + VT	Adjusted mean difference (SLR − SLR + VT)	95% CI
**Eye–hand coordination (EHC)**	39.130 (1.020)	38.544 (1.020)	+0.586	−2.375 to 3.548
**Visual memory (accuracy)**	66.048 (7.703)	54.235 (7.974)	+11.813	−11.003 to 34.630
**Spatial (accuracy)**	69.561 (5.650)	65.041 (5.850)	+4.520	−12.267 to 21.306
**Peripheral (accuracy)**	88.913 (6.234)	81.087 (6.234)	+7.826	−10.830 to 26.483
**Central/peripheral (accuracy)**	39.712 (3.643)	41.422 (3.643)	−1.710	−12.494 to 9.075
**Eye movement (reaction time)**	0.524 (0.018)	0.520 (0.018)	+0.004	−0.050 to 0.058
**TUG (time)**	6.435 (0.124)	6.566 (0.124)	−0.131	−0.490 to 0.228

Adjusted post-intervention means (SE) for each group are shown. Between-group differences were estimated using ANCOVA with group (SLR vs. SLR + VT) as a fixed factor and the corresponding baseline value as a covariate.

For eye–hand coordination, which was treated as a key exploratory visual-function outcome (completion time, lower is better), the adjusted mean difference (SLR − SLR + VT) was + 0.586 (95% CI −2.375 to 3.548). The adjusted mean difference for visual memory (accuracy, higher is better) was + 11.813 (95% CI −11.003 to 34.630); spatial cognition (accuracy), + 4.520 (95% CI −12.267 to 21.306); peripheral perception (accuracy), + 7.826 (95% CI −10.830 to 26.483); central/peripheral perception (accuracy), −1.710 (95% CI −12.494 to 9.075); eye movement reaction time (lower is better), + 0.004 (95% CI −0.050 to 0.058); and the Timed Up and Go test (TUG; completion time, lower is better), −0.131 (95% CI −0.490 to 0.228). No adverse events or unintended effects related to the intervention occurred.

## Discussion

In this exploratory pilot randomized trial, no clear baseline-adjusted between group differences were observed in post-intervention visual-function or physical function outcomes (Table 2). Because both programs were time-matched and the sample size was small, these estimates should be interpreted cautiously, as they are expected to be imprecise. Importantly, the direction of point estimates suggested that VT may have had small favorable effects on selected outcomes conceptually aligned with repeated eye- movement practice (e.g., Slightly shorter EHC completion time and slightly higher central/peripheral perception accuracy), although confidence intervals were wide and included no difference. Thus, rather than concluding that VT is ineffective, the present findings indicate that any VT-specific benefit of this simple program, if present, was not detected with adequate precision in this pilot trial. The results should therefore be considered hypothesis-generating and informative for the design of a fully powered trial.

The intervention was well tolerated by older adults, with no adverse events reported and no dropouts during the intervention period. The VT component consisted of simple horizontal, vertical, and near–far (anteroposterior) eye movements performed in a seated position. Accordingly, mechanistic interpretations should be limited to processes plausibly engaged by repeated eye-movement practice (e.g., oculomotor control and extraocular muscle function), rather than higher-order cognitive processes.

Several factors may explain why clear baseline-adjusted differences between programs were not observed. First, the VT component was low-dose and non-progressive, without increases in intensity or task complexity, which may have been insufficient to influence outcomes requiring substantial visuomotor integration or cognitive engagement (e.g., visual memory or spatial cognition). Second, the outcome battery included tasks that varied in their specificity to the training stimulus; larger effects may be more likely when the training and outcome share common task demands (task specificity). Third, because both groups received active training and were repeatedly assessed, non-specific influences such as attention, engagement, and familiarization with testing may have affected outcomes in both groups, potentially reducing detectable between-program differences. Future trials should therefore incorporate time- and attention-matched control conditions and a VT program that includes progression and more task-specific components.

Potential mechanisms remain speculative because neuromuscular and neurophysiological measures were not collected. Nevertheless, eye-movement training may indirectly activate spatial working memory [21]. Furthermore, the ability to utilize peripheral vision is a critical function directly related to fall risk and hazard detection [22]. Repeated eye-movement exercises may stimulate extraocular muscles and support maintenance of eye-movement speed and control, which are known to decline with aging, and such activation could contribute to general changes in visual task performance; however, these interpretations require direct verification in future studies [23].

Consistent with the simplicity of the VT component, we did not observe clear improvements in outcomes that typically require substantial visuomotor integration or cognitive engagement (e.g., EHC, visual memory, and spatial cognition). These functions are often targeted by training paradigms that explicitly require coordinated visuomotor responses or higher task complexity. The absence of clear changes in the present trial may therefore reflect the non-progressive, low-complexity VT protocol. Previous studies suggest that training designed to synchronize eye and hand movements can engage neural systems for visuomotor integration and may improve eye–hand coordination and related functions [24,25].

Any small changes in visual-function outcomes observed in this study may instead have occurred indirectly through repeated eye-movement tasks, which could promote visuomotor integration and reactivation of extraocular muscles [26–28].

Importantly, the present study was not designed to directly evaluate fall prevention. Although visual function is relevant to mobility and environmental interaction, the intervention content and outcome measures were insufficient to draw conclusions regarding effects on fall risk or gait performance. Future studies employing attention-matched control conditions, balance-related outcomes, and participants at higher risk of falls are needed to clarify the clinical relevance of VT within fall-prevention programs. Future research should clarify whether VT contributes to fall prevention and gait improvement in older adults, thereby strengthening its clinical applicability in rehabilitation and preventive programs. Reduced visual search ability has been reported in older adults and may be compensated by eye-movement training [29]. Although sensory integration and motor control decline with age, they remain trainable [30]. Furthermore, visual interventions can improve postural control and gait [31–34]. These findings underscore the importance of further investigation.

These findings should be interpreted with caution, given several methodological limitations, including the small sample size, lack of blinding, reliance on self-reported visual acuity, potential non-specific effects due to the control condition, sex imbalance, and the non-progressive nature of the VT protocol. First, visual acuity was based on self-report rather than objective measurement, although all participants reported vision sufficient for daily life. Second, most participants were female (27 women and 3 men), limiting the examination of sex-related differences; future studies should recruit a more balanced sample. Third, simple randomization without allocation concealment was used, and outcome assessors and data analysts were not blinded to group allocation, which may have introduced selection, performance, and detection bias. Fourth, the VT program was low-dose and non-progressive, without increases in intensity or task complexity, which may have limited its potential effects on more complex visuomotor or cognitive outcomes. Fifth, the outcome battery included tasks that varied in their specificity to the training stimulus; larger effects might be expected when the training and outcome share common task demands (task specificity). Finally, because both groups received active training and were repeatedly assessed, non-specific influences such as attention, engagement, and familiarization with testing may have affected outcomes in both groups, potentially reducing detectable between-program differences. Future trials should therefore incorporate time- and attention-matched control conditions, objective visual assessments, blinded outcome assessment where feasible, and VT programs with greater progression and task specificity.

## Conclusion

In this exploratory pilot randomized trial of community-dwelling older adults, no clear baseline-adjusted between-group differences were observed in post-intervention visual-function or physical-function outcomes. Given the pilot sample size, estimates were imprecise and confidence intervals were wide; therefore, these findings should not be interpreted as definitive evidence of no effect. The intervention was completed by all participants without reported adverse events, and the results should be considered hypothesis-generating. Future adequately powered trials using time- and attention-matched control conditions and clinically meaningful endpoints are warranted to clarify the potential clinical value of VT in older adults.

## Supporting information

S1 File CONSORT 2010 checklist. (PDF)

S2 File English translation of the main points of the study protocol. (PDF)

S3 File Original Japanese version of the study protocol. (PDF)

S4 File Full English translation of the original study protocol. (PDF)
