# Supplementary material for: Effect of vision training on visual function in older adults: A pilot randomized trial
Source: PLoS One. 2026 Aug 24;21(8):e0356206. doi: 10.1371/journal.pone.0356206 (PMC13502553; doi:10.1371/journal.pone.0356206)
Supplement: Supplementary file 1 [file pone.0356206.s001.pdf]

The CONSORT reporting checklist

For checking that reports of randomised trials can be understood and used by everyone

> ⓘ Note
>
> If you have not used a reporting guideline before, read about how and why to use them and check whether CONSORT is the most applicable reporting guideline for your work.
>
> Reporting guidelines are most useful when used early in research. When writing a manuscript or application, consider using the Full Guidance where you'll see explanations and examples for each item.
>
> After writing, demonstrate adherence by completing this checklist:
>
> 1. Specify where each item is described (see Note 1).
> 2. Cite this checklist (See Note 2).
> 3. Include your completed checklist as a supplement when submitting to a journal so that future readers can use it to find information.

	Item Description	Location (or reason for not reporting)
**Title and Abstract**		
1a. Title	Identification as a randomised trial.	P1 Line1-2
1b. Structured Abstract	Structured summary of the trial design, methods, results, and conclusions.	P2 Line18-39; Abstract
**Open Science**		
2. Trial Registration	Name of trial registry, identifying number (with URL) and date of registration.	P7 Line112; Methods-Experimental Procedure
Protocol and statistical analysis plan	Where the trial protocol and statistical analysis plan can be accessed.	P7 Line112<br>Protocol and Statistical Analysis Plan available at UMIN-CTR (UMIN000050796).
4. Data sharing	Where and how the individual de-identified participant data (including data dictionary), statistical code and any other materials can be accessed.	Not publicly available due to privacy restrictions; de-identified participant data and statistical code are available from the corresponding author upon reasonable request.
5. Funding and Conflicts of Interest		
5a. Funding	Sources of funding and other support (eg, supply of drugs), and role of funders in the design, conduct,	JSPS KAKENHI (JP23K16700); the funder had no role in the

	analysis, and reporting of the trial.	study.
5b. Conflicts of interest	Financial and other conflicts of interest of the manuscript authors.	The authors declare no conflicts of interest.
**Introduction**		
6. Background and rationale	Scientific background and rationale.	P3-5 Line41-75 Introduction
7. Objectives	Specific objectives related to benefits and harms.	P5 Line76-85 Introduction
**Methods**		
8. Patient and public involvement	Details of patient or public involvement in the design, conduct and reporting of the trial.	Patients and the public were not involved in the design, conduct, or reporting of this trial.
9. Trial Design	Description of trial design including type of trial (eg, parallel group, crossover), allocation ratio, and framework (eg, superiority, equivalence, non-inferiority, exploratory).	P.6 Lines 93–104 Methods-Participants; parallel-group randomized trial with 1:1 allocation.
10. Changes to trial protocol	Important changes to the trial after it commenced including any outcomes or analyses that were not pre-specified, with reason.	No changes to the protocol after trial commencement.
11. Trial Setting	Settings (eg, community, hospital) and locations (eg, countries, sites) where the trial was conducted.	P5-6 Lines 88–92 Methods-Participants; conducted among community-dwelling older adults.
12. Eligibility Criteria		
12a. Participants	Eligibility criteria for participants.	P5-6 Lines 88–96 Methods-Participants; eligibility criteria described.
12b. Other	If applicable, eligibility criteria for sites and for individuals delivering the interventions (eg, surgeons, physiotherapists).	Not applicable; no site-level or provider-level eligibility criteria.
13. Intervention and comparator	Intervention and comparator with sufficient details to allow replication. If relevant, where additional materials describing the intervention and comparator (eg, intervention manual) can be accessed.	P7-12 Lines 116–205 Methods-Measures; intervention and comparator procedures described.
14. Outcomes	Prespecified primary and secondary outcomes, including the specific measurement variable (eg, systolic blood pressure), analysis metric (eg, change from baseline, final value, time to event), method of aggregation (eg, median, proportion), and time point for each outcome.	P8 Lines 134–141 Methods-Primary and secondary outcomes; primary and secondary outcomes defined.
15. Harms	How harms were defined and assessed (eg, systematically, non-systematically).	No harms were observed or specifically assessed during the

		trial.
16. Sample Size		
16a. How sample size was determined	How sample size was determined, including all assumptions supporting the sample size calculation.	P6 Lines 92–96<br>Methods-Participants; sample size based on feasibility considerations.
16b. Interim analyses and stopping criteria	Explanation of any interim analyses and stopping guidelines.	No interim analyses or stopping criteria were planned.
17. Randomisation		
17a. Sequence Generation	Who generated the random allocation sequence and the method used.	P6 Lines 98–103<br>Methods- Experimental Procedure; simple randomization using a random number table.
17b. Type of Randomisation	Type of randomisation and details of any restriction (eg, stratification, blocking, and block size).	P6 Lines 98–103<br>Methods- Experimental Procedure; Simple randomization without blocking or stratification.
18. Allocation concealment mechanism	Mechanism used to implement the random allocation sequence (eg, central computer/telephone; sequentially numbered, opaque, sealed containers), describing any steps to conceal the sequence until interventions were assigned.	P6 Lines 98–103<br>Methods- Experimental Procedure; no allocation concealment was used.
19. Implementation	Whether the personnel who enrolled and those who assigned participants to the interventions had access to the random allocation sequence.	P6 Lines 98–103<br>Methods- Experimental Procedure; investigator generated the sequence and assigned participants.
20. Blinding		
20a. Who was blinded	Who was blinded after assignment to interventions (eg, participants, care providers, outcome assessors, data analysts).	P6 Lines 104–105<br>Methods- Experimental Procedure; not blinding of participants, assessors, or analysts.
20b. How blinding was achieved	If blinded, how blinding was achieved and description of the similarity of interventions.	P6 Line104-105 Blinding was not performed due to the nature of the intervention.
21. Statistical methods		
21a. Comparing groups	Statistical methods used to compare groups for primary and secondary outcomes, including harms.	P13 Lines 218–226 Methods-Statistical analysis; ANCOVA used to compare groups with

		baseline values as covariates.
21b. Definition of who is included in each analysis	Definition of who is included in each analysis (e.g., all randomised participants), and in which group.	P13 Line219-226; Methods-Statistical analysis; All participants with complete post-intervention data were included in the analysis.
21c. Missing Data	How missing data were handled in the analysis.	No missing data imputation was performed; analyses were conducted on complete cases.
21d. Additional Analyses	Methods for any additional analyses (eg, subgroup and sensitivity analyses), distinguishing pre-specified from post hoc.	No additional analyses (e.g., subgroup or sensitivity analyses) were conducted.
22. Participant flow, including flow diagram		
22a. Participant Numbers	For each group, the numbers of participants who were randomly assigned, received intended intervention, and were analysed for the primary outcome.	P13 Results; Participant flow and Fig. 1.
22b. Losses and exclusions	For each group, losses and exclusions after randomisation, together with reasons.	P13 Results; Fig. 1 (flow diagram) and Participant flow paragraph.
23. Recruitment		
23a. Dates	Dates defining the periods of recruitment and follow-up for outcomes of benefits and harms.	P6 Line92-96; Methods Participants — recruitment 15 Jan–31 Mar 2024; baseline/interventions Apr–Aug 2024; post-intervention assessments immediately after the 8-week program.
23b. Reasons for stopping recruitment	If relevant, why the trial ended or was stopped.	P6 Line92-96; Recruitment ended as planned when the target sample size (n=30) was reached; no early stopping.
24. Intervention and comparator delivery		
24a. As Administered	Intervention and comparator as they were actually administered (eg, where appropriate, who delivered the intervention/comparator, whether participants adhered, whether they were delivered as intended (fidelity)).	P11 Line189-216; Methods; Intervention SLR vs SLR+VT; 3 sessions/week for 8 weeks; exercise procedures and posture described.
24b. Concomitant Care	Concomitant care received during the trial for each group.	Not reported; no specific concomitant care described during the trial.

25. Baseline Data	A table showing baseline demographic and clinical characteristics for each group.	P13-14 Line229-233<br>Results; Table 1 — baseline demographic and clinical characteristics by group.
26. Numbers analysed, outcomes, and estimation	For each primary and secondary outcome, by group:<br>• the number of participants included in the analysis.<br>• the number of participants with available data at the outcome time point.<br>• result for each group, and the estimated effect size and its precision (such as 95% confidence interval).<br>• for binary outcomes, presentation of both absolute and relative effect size.	P14-16 Line234-254<br>Results; Outcomes — ANCOVA-adjusted between-group estimates with 95% CIs reported for primary and secondary outcomes (see Tables 2–3 / Figures as applicable).
27. Harms	All harms or unintended events in each group.	P13 Line228<br>Results; Harms/Safety: No adverse events were observed during the 8-week intervention period.
28. Ancillary Analyses	Any other analyses performed, including subgroup and sensitivity analyses, distinguishing pre-specified from post hoc.	Not applicable; no subgroup or sensitivity analyses were conducted.
**Discussion**		
29. Interpretation	Interpretation consistent with results, balancing benefits and harms, and considering other relevant evidence.	P16-17 Lines 256–274<br>Discussion; interpretation of findings considering benefits, potential harms, precision, and alignment with prior evidence.
30. Limitations	Trial limitations, addressing sources of potential bias, imprecision, generalisability, and, if relevant, multiplicity of analyses.	P19-20 Lines 315–328<br>Discussion; limitations including measurement approach, sample characteristics, non-progressive intervention dose, task specificity, and potential non-specific influences.

1 How to specify where content is

Tell the reader where they can find information. E.g.,

- Results; paragraph 2
- Methods, Participants; paragraphs 1 & 2.
- Table 3

- Supplement B, para. 4

If you have chosen not to describe an item, explain why. You can do this in the checklist, or as a note below it.

You can describe items in the article body, or in tables, figures, or supplementary materials, and should prioritize items you feel are most important to your intended audience. The order of items in your manuscript does not need to match the order of items in this checklist. You can decide how best to structure your work.

2 How to cite

Describe how you used CONSORT at the end of your Methods section, referencing the resources you used e.g.,

> 'We used the CONSORT reporting guideline(1) to draft this manuscript, and the CONSORT reporting checklist(2) when editing, included in supplement A'

If you use a reporting checklist, remember to include it as a supplement when publishing so that readers can easily find information and see how you have interpreted the guidance.

1. Hopewell S, Chan AW, Collins GS, Hróbjartsson A, Moher D, Schulz KF, et al. CONSORT 2025 statement: Updated guideline for reporting randomised trials. The BMJ [Internet]. 2025 Apr;389:e081123. Available from: https://www.ncbi.nlm.nih.gov/pmc/articles/PMC11995449/

2. Hopewell S, Chan AW, Collins GS, Hróbjartsson A, Moher D, Schulz KF, et al. The CONSORT reporting checklist. In: Harwood J, Albury C, Beyer J de, Schlüssel M, Collins G, editors. The EQUATOR network reporting guideline platform [Internet]. The UK EQUATOR Centre; 2025. Available from: https:/resources.equator-network.org/reporting-guidelines/consort/consort-checklist.docx
