# Supplementary material for: Effect of vision training on visual function in older adults: A pilot randomized trial
Source: PLoS One. 2026 Aug 24;21(8):e0356206. doi: 10.1371/journal.pone.0356206 (PMC13502553; doi:10.1371/journal.pone.0356206)
Supplement: Supplementary file 2 [file pone.0356206.s002.pdf]

1. Title of the Study

Development of a Vision Training Method to Acquire Safe Obstacle-Crossing Movements During Walking

---

2. Study Organization (Research Team at Time of Ethical Approval)

- **Principal Investigator:** Daisuke Sudo, Assistant Professor
  School of Health Sciences at Odawara, International University of Health and Welfare
  Roles: Study oversight, data collection, data analysis
- **Co-investigator:** Yusuke Maeda, Associate Professor
  Roles: Data collection, data analysis, research supervision
- **Co-investigator:** Masato Hosokawa, Assistant Professor
  Roles: Data collection, data analysis

(Note: This list reflects the team at the time of ethical approval and may differ from authors listed in the manuscript.)

---

3. Study Period

From the date of IRB approval to **March 31, 2027**.

---

4. Purpose and Significance

This study aims to develop a vision training method that improves visuomotor coordination in older adults during obstacle-crossing movements, thereby contributing to fall prevention. Prior research by the investigator has shown that older adults with high fall risk demonstrate abrupt reductions in step length and trunk rotation before passing through narrow spaces, likely due to decreased visual function and reliance on central vision.

The study seeks to determine whether applying vision-training approaches—traditionally used in athletes—can improve visual function and movement coordination in older adults.

---

5. Study Methods

Design

Interventional study using 3D motion analysis and visual function assessment systems.

Participants

- Older adults: 40
- Younger adults: 20 (for reference measurements)

Randomization

Older adults will be randomly assigned (using random number tables) to one of two groups:

1. **Strength Training Group** (lower-limb and trunk strengthening)
2. **Vision + Strength Training Group** (strength training plus vision training)

Intervention

Training 3 times per week for 8 weeks.
Vision training follows the methods recommended by the Japan Sports Vision Association and focuses on:

- peripheral awareness
- visuomotor coordination
- spatial recognition
- ocular motor control
  Training will be conducted in a seated position.

Outcome Measures

Primary outcomes:

- Step length
- Obstacle fixation time

Secondary outcomes:

- Joint angles (hip, knee, ankle)

- Joint moments
- Obstacle–toe clearance
- Visual function measures (hand-eye coordination, peripheral awareness, memory, etc.)

Statistical Analysis

Paired t-test for pre–post comparisons. Dropouts will be excluded from analysis.

---

6. Sample Size Justification

Based on an effect size of 0.5, $\alpha = 0.05$, and power = 0.8, with reference to previous studies, 40 older adults were deemed appropriate.

---

7. Inclusion / Exclusion Criteria

Inclusion criteria

- No gait-impairing neurological or orthopedic issues
- No cognitive impairment
- Independent ambulation

Exclusion criteria

- Significant visual impairment
- Pain during walking
- Use of walking aids

---

8. Informed Consent

Written informed consent will be obtained from all participants, with verbal explanation provided beforehand.
Students will be informed that participation (or refusal) has no effect on academic standing.

Older adults will be informed that refusal has no effect on participation in community fall-prevention programs.

---

9. Data Handling and Confidentiality

- Personal identifiers (name, age) will be collected only as needed.
- Pseudonymized data will be managed securely on password-protected systems and locked storage.
- Data will be retained for **10 years** after publication.
- No data will be shared with external institutions.

---

10. Risks and Benefits

Risks:

- Potential risk of falls during movement tasks
- Physical burden of measurements (approx. 60 minutes)

Benefits:

- Possible improvement in visuomotor coordination through vision training

Safety measures include:

- 2 physical therapists present during measurement
- Immediate cessation of tasks if signs of fatigue or instability appear

---

11. Trial Registration

Registered in UMIN Clinical Trials Registry
UMIN000050796

---

12. Publication Plan

Results will be submitted for publication after study completion.
