# Supplementary material for: Effect of vision training on visual function in older adults: A pilot randomized trial
Source: PLoS One. 2026 Aug 24;21(8):e0356206. doi: 10.1371/journal.pone.0356206 (PMC13502553; doi:10.1371/journal.pone.0356206)
Supplement: Supplementary file 3 [file pone.0356206.s003.pdf]

病院・施設・大学・教職員用

(受付番号　　－　　－　　)

# 国際医療福祉大学

# 研究計画書

Version0.2

(西暦)2023 年 5 月 11 日

| | | |
|---|---|---|
| 研究責任者 | 施　　設： | 国際医療福祉大学 |
| | 所　　属： | 小田原保健医療学部理学療法学科 |
| | 職　　名： | 助教 |
| | 氏　　名： | 須藤　大輔 |
| | 電話番号　：　0465-21-6724 | |
| | Mail:　d.sudo@iuhw.ac.jp | |
| 所属長 | 職　　名： | 教授 |
| | 氏　　名： | 久保　晃 |

**本研究計画書については、提出前に所属長に報告し了解を得ております。　■はい　□いいえ**

(上記は、「はい」にチェックがあることが必須です。虚偽の申告が発覚した場合、発覚した日より 2 年間倫理審査を受け付けられません。)

1．研究の名称
指針第7(1)①

歩行中の安全な障害物跨ぎ動作獲得のためのビジョントレーニング手法の開発

2．研究の実施体制
(研究機関の名称及び研究者等の氏名を含む。)
指針第7(1)②

| 研究者氏名 | 研究機関名 | 所属・職名 | 資格・役割・分担など |
|---|---|---|---|
| 須藤大輔 | 国際医療福祉大学 | 小田原保健医療学部　理学療法学科<br>助教 | 研究責任者<br>データ計測<br>データ解析 |
| 前田佑輔 | 国際医療福祉大学 | 小田原保健医療学部　理学療法学科<br>准教授 | データ計測<br>データ解析<br>研究指導 |
| 細川真登 | 国際医療福祉大学 | 小田原保健医療学部　理学療法学科<br>助教 | データ計測<br>データ解析 |

①研究機関・共同研究機関における研究者等

②研究協力機関、その他倫理審査の対象とならない機関において当該研究に係る者
該当なし

③研究に関わる業務の一部外注先（外注する業務内容と外注先を記載）
該当なし

3．研究実施期間
指針第7(1)④

承認日以降 ～ 西暦 2027 年 3 月 31 日

4．研究の目的と意義
指針第7(1)③

(1)研究の目的及び意義
転倒予防目的に運動機能向上を図っても十分な効果が得られないことが問題となっている。高齢者が安全に障害物を回避するには、視覚に基づいて自己と環境を知覚し、姿勢や歩容を調整することが重要であるが、転倒予防のエビデンスとしては未構築である。申請者は自己と環境を知覚する課題として隙間を通過する動作の研究を行い、転倒リスクの高い高齢者は隙間通過の直前に歩幅の急激な縮小と骨盤回旋をすることを明らかにした。その原因として視機能の低下による中心視への依存が影響していると考え、今回、安全な障害物回避動作獲得に向けビジョントレーニングの効果を明らかにすることを目的に行う。アスリートに対し行われていた視線へのアプローチを高齢者に取り入れることで、視覚と身体運動の協調性が向上し、歩幅の急激な変動を防ぐことができ、転倒予防に貢献できると考える。

(2)予想される医学上の貢献及び意義
転倒発生の多い障害物跨ぎ動作については、視覚情報の取り方に原因があると考え、身体と環境の関係を調整する際に重要な視線へのアプローチ方法の開発と予防トレーニング効果を明らかにし、地域の転倒予防アプローチの一助とする。

5. 研究の方法
指針第7(1)④

(1)研究方法の概要
40 名の高齢者を乱数表を用いた無作為割り付けにより 2 群に分ける。一方を下肢、体幹筋力強化を中心としたトレーニング群、もう一方を下肢、体幹筋力強化＋ビジョントレーニング群とし、トレーニング前後での障害物跨ぎ動作時の股関節、膝関節、足関節角度、股関節、膝関節、足関節モーメント、障害物注視時間、歩幅、踏切時の障害物－足尖距離、視機能の変化を比較する。視機能評価は日本スポーツビジョン協会で推奨されている v-training を使用し視力、眼と手/身体の協応動作、瞬間視記憶、周辺視野の感知力、空間認識能力、眼球運動を評価する。その後、週 3 回 8 週間の各トレーニングを実施し、再び隙間通過動作の三次元動作分析、視線計測、視機能評価を行う。比較対象のため若年者 20 名の隙間通過動作の計測、視線計測を実施する。ビジョントレーニングの方法は日本スポーツビジョン協会で推奨されているものを採用し、周辺部の感知力向上を目的に以下の眼球を動かす運動を行う。めまいやふらつきにより転倒することを防ぐためにビジョントレーニングは座位にて週 3 回を 8 週間行う。その後トレーニング前後で対応のある t 検定を行う。脱落例については解析データから除外する。

(2)研究のデザイン
国際医療福祉大学単独・三次元動作分析と視機能評価機器を使用したビジョントレーニングの効果検証を目的とする介入研究

(3)当該研究で用いる医薬品・医療機器・治療方法・観察方法等
該当なし

(4)評価項目及び評価方法
1)主要評価項目　歩幅、障害物注視時間

2)副次評価項目　股関節角度、膝関節角度、足関節角度

(5)統計解析方法

40 名の高齢者を乱数表を用いた無作為割り付けにより 2 群に分ける。一方を下肢、体幹筋力強化を中心としたトレーニング群、もう一方を下肢、体幹筋力強化＋ビジョントレーニング群とし、トレーニング前後での障害物跨ぎ動作時の股関節、膝関節、足関節角度、股関節、膝関節、足関節モーメント、障害物注視時間、歩幅、踏切時の障害物－足尖距離、視機能の変化を比較する。その後、週 3 回 8 週間のビジョントレーニングを実施し、再び隙間通過動作の三次元動作分析、視線計測、視機能評価を行う。比較対象のため若年者 20 名の隙間通過動作の計測、視線計測を実施する。統計方法はトレーニング前後で対応のある t 検定を行う。脱落例については解析データから除外する。

(6)予定する研究対象者数

若年者 20 人

高齢者 40 人

(7)対象者数の設定根拠

サンプルサイズを効果量 0.5、αエラー0.05、検定力 0.8 での算出と先行研究の傾向を加味して、検討した結果、目標とする対象者(高齢者)は 40 名とする。

参考文献:Efficacy of a Digital Sports Vision Training Program for Improving Visual Abilities in Collegiate Baseball and Softball Athletes, 2021

Testing the efficacy of vision training for presbyopia: alternating-distance training does not facilitate vision improvement compared to fixed-distance training, 2022

(8)研究対象者の研究参加予定期間(症例登録期間およびその後の観察期間等)

初回計測日、トレーニング実施期間週 3 回 8 週間、最終計測日

(9)当該研究に用いる試料・情報とそれらの収集スケジュール

初回計測日:課題動作計測(3 次元動作分析装置による障害物跨ぎ動作の計測)
　　　　　　身体機能評価(転倒経験、片脚立位時間、Timed up and go test)
　　　　　　視機能評価(視機能評価眼と手/身体の協応動作、瞬間視記憶、
　　　　　　周辺視野の感知力、空間認識能力、眼球運動)
トレーニング実施期間:週 3 回 8 週間実施
最終計測日:課題動作計測(3 次元動作分析装置による障害物跨ぎ動作の計測)
　　　　　　身体機能評価(転倒経験、片脚立位時間、Timed up and go test)
　　　　　　視機能評価(視機能評価眼と手/身体の協応動作、瞬間視記憶、
　　　　　　周辺視野の感知力、空間認識能力、眼球運動)

(10)研究計画書・同意説明文書等の変更

本研究の研究計画書や同意説明文書等の変更または改訂を行う場合は、あらかじめ倫理審査委員会の承認を得て実施する。

(11)研究の中止基準と研究参加者への対応
1)研究中止時の対応
研究責任者または研究分担者は、以下の理由で個々の研究対象者について、研究継続が不可能と判断した場合には、当該研究対象者について説明する。
2)中止基準
1. 研究対象者から同意の撤回があったとき
2. 被験者から体調不良の訴えがあったとき
3. 下記(12)により本研究全体が中止されたとき
4. その他の理由により、研究責任者が研究の中止が適当と判断した場合

中止になった対象者のデータは速やかに削除し、撤回時点までのデータは使用しない。

(12)研究参加者に対する研究終了後の対応
研究終了後に参加者からの問い合わせがあった場合には、速やかに情報を開示し問い合わせに対して回答および説明を実施する。

(13)他機関への試料・情報の提供
該当なし

6. 研究対象者の選定方針
指針第7(1)⑤

(1)対象者の概要
対象数: 60 名(若年者 20 名、高齢者 40 名)

性別:男女
年齢層: 20 歳～ 75 歳

□①未成年者が含まれるか
□はい、含まれます
■いいえ、含まれません
⇒「はい」の場合、以下の内容について下記に記載
代諾者等の選定方針
代諾者への説明事項
研究対象者は(未成年、青年であってICを与える能力を欠く、死者)
未成年であっても当該研究対象者からインフォームド・コンセントを受ける(下記説明参照)

□②本学もしくは本学以外の大学生、大学院生が含まれる
■はい、含まれます
□いいえ、含まれません

□③同意能力が不十分な成年者が含まれる
□はい、含まれます
■いいえ、含まれません
⇒「はい」の場合、以下の内容について検討し下記に記載
本研究に①～③に該当する対象者の参加が必要不可欠な理由と必要な対応:
②について、計測機器が大学に設置されているため、外部に出向いて計測することが困難であるため、小田原保健医療学部理学療法学科の学生に被験者依頼をする。

(2)研究対象者の選定方針

1）選択基準

①計測日の時点で運動麻痺や歩行に支障が出る関節可動域制限や疼痛などがない者

②認知機能の低下がなく評価および計測内容が理解できる者

③歩行が自立している者

2）除外基準

①日常生活に支障をきたす視覚障害の者

②歩行時に疼痛を訴える者

③歩行補助具を使用している者

(3) 対象者募集のプロセス

若年者は小田原保健医療学部理学療法学科の学生に被験者募集を募り、希望者を対象に計測を行う。

高齢者は申請者の関わっている任意団体の転倒予防教室で募集する。

(4) 研究開始後に対象者を除外する条件とその試料・情報の取り扱い
・研究登録後（ランダム割付後）に同意撤回が表明された場合
・研究登録後（ランダム割付後）に選択/除外基準からの逸脱が発覚した場合
・研究登録後（ランダム割付後）にプロトコル逸脱が発覚した場合

同意撤回の場合はその症例の試料・情報はすべて破棄する

(5) 大学生、大学院生を対象とする場合の配慮

■①研究者の担当する科目について、研究への参加の有無が学業成績や単位取得に影響を与えない旨を募集要領に明記している

■②申請者と同じ研究室に所属する学生は含まれていない

■③研究への参加を拒んでも、学業成績や単位取得に影響を与えない旨を説明文書に明記している

■④参加の同意書は、研究についての説明を十分におこなった後、日を改めて提出してもらう

□⑤その他の配慮（　　　　　　　　　　　　　　　　）

| | |
|---|---|
| ７．研究の科学的合理性の根拠<br>指針第7(1)⑥ | 高齢者が障害物に躓く要因を三次元動作解析装置および視機能評価を用いて分析し、ビジョントレーニングによって身体運動や視機能が変化するのかを検証する。<br>研究期間は５年を予定している。 |

| | |
|---|---|
| 8．インフォームド･コンセントを受ける手続等<br>指針第7(1)⑦ | (1)手続き方法<br>文書を用いて説明･同意を取得する。学部生を対象とする場合には、同意を得る際には、仮に同意しなかった場合にも成績には一切影響はない旨を説明する。<br>文書･口頭による説明は計測開始前に行う。<br>高齢者にも同様に文書を用いて説明･同意を取得する。同意を得る際には、仮に同意しなかった場合にも転倒予防教室の活動には一切影響はない旨を説明する。<br>(2)同意取得の具体的方法<br>1)説明の方法:文書を添えて口頭にて説明する<br>2)説明の実施者:研究責任者<br><br>(3)同意の撤回等<br>1)研究実施によって既に取得済みの試料･情報について使用の停止･廃棄等<br>同意撤回の意向がある場合には速やかに中止し、収集データは破棄する。<br>2)他機関への提供した試料･データの差し止め等<br>他機関への試料･データの提供はない<br>3)研究が実施又は継続されることに同意した場合であっても随時これを同意を撤回できる |
| 9．個人情報等の取扱い(匿名化する場合にはその方法、匿名加工情報又は非識別加工情報を作成する場合にはその旨を含む。)<br><br>指針第18(1) | (1)個人情報の取扱い<br>この研究で個人情報を取り扱う　■はい　□いいえ<br><br>⇒「いいえ」→(2)へ　「はい」→(3), (4)へ<br><br>(2)扱う情報の種類<br>□仮名加工情報(既に作成されているものに限る. 作成元:○○○)<br>□匿名加工情報(既に作成されているものを含む. 作成元:○○○)<br>□個人関連情報<br>(具体的な内容:　　　　　　　　　　　　　　　　　　　　　　　　)<br>□上記のいずれも取り扱わない<br><br>(3)収集する個人情報<br>■①氏名、生年月日、その他の記述等に記載され、もしくは記録され、特定の個人を識別できるもの<br>具体的な内容:氏名、年齢<br>個人情報の利用目的:基本情報収集のため<br>□②音声、動作その他の方法を用いて表された一切の事項により特定の個人を識別できるもの<br>具体的な内容:<br>個人情報の利用目的:<br>□③個人識別符号が含まれるもの<br>具体的な内容:<br>個人情報の利用目的:<br>□④死者について特定の個人を識別することができる情報を含めたもの<br>具体的な内容:<br>個人情報の利用目的:<br>…………………………………………………………………………<br>□要配慮個人情報を含む<br>具体的な内容:<br>個人情報の利用目的: |

(4)仮名加工情報あるいは匿名加工情報の作成
□作成しない

■仮名加工情報を作成する
担当者名:須藤大輔
所属:小田原保健医療学部理学療法学科
資格:研究責任者
対応表の管理方法:
■外部と切り離された PC を使用して外部記憶媒体に保存し鍵をかけて厳重に保管する
■紙媒体に記録し、鍵をかけて厳重に保管する
■その他
具体的に:本研究に係わるすべての研究者は、「ヘルシンキ宣言」および「人を対象とする生命科学・医学系研究に関する倫理指針」を遵守して実施する。
研究実施に係る試料・情報を取扱う際は、研究対象者の個人情報とは無関係の番号を付して管理し、研究対象者の秘密保護に十分配慮する。研究の結果を公表する際は、研究対象者を特定できる情報を含まないようにする。また、研究の目的以外に、研究で得られた研究対象者の試料・情報を使用しない。

□匿名加工情報を作成する
担当者名:
所属:
資格:
加工方法の概略:
具体的な内容:

| | |
|---|---|
| 10. 研究対象者に生じる負担並びに予測されるリスク及び利益、これらの総合的評価並びに当該負担及びリスクを最小化する対策<br>指針第7(1)⑨ | (1)研究対象者に生じる負担<br>三次元動作解析や視機能の計測は実施手順を確認し、なるべく拘束時間を少なくできるように配慮する。データ収集時間:60 分程度<br>被験者の移動に伴う交通費は実費支払いする。<br><br>(2)予測されるリスク<br>本研究は介入研究であり、介入前後の身体機能評価において片脚立位時間計測や障害物を跨ぐ動作の計測の際に転倒のリスクがある。<br><br>(3)予測される利益<br>本研究で実施するビジョントレーニングは、その結果により身体運動の変容という利益が生じることが期待される。<br><br>(4)総合的評価並びに当該負担及びリスクを最小化する対策<br>動作計測および身体機能評価(片脚立位、Timed up and go test)の際は、転倒防止策として複数名で対応し、ふらつきが生じた際にすぐに補助できる位置で評価を行う。研究対象者の体調をよく確認し、不調であれば、計測や評価を中止する。<br><br>危害の発生または危害の程度を最小にするためにとるべき体制:計測の際は必ず理学療法士 2 名体制で実施する。 |

| | |
|---|---|
| | 危害が発生した場合の対応:理学療法学科教員での初期対応を行い、対応困難と判断した場合には学部長(医師)、看護学科教員の応援を要請する。医療機関での治療が必要となった場合には速やかに救急要請する。 |
| 11. 侵襲(軽微な侵襲を除く。)を伴う研究の場合<br>指針第7(1)⑳<br>指針第7(1)㉑ | □ 該当しない |
| | －1 重篤な有害事象が発生した際の対応 |
| | 理学療法学科教員での初期対応を行い、対応困難と判断した場合には学部長(医師)、看護学科教員の応援を要請する。医療機関での治療が必要となった場合には速やかに救急要請する。 |
| | □ 該当しない |
| | －2 当該研究によって生じた健康被害に対する補償の有無及びその内容 |
| | 補償・賠償に関しては被害が起こった時には自分の個人保険(理学療法士協会賠償責任保険)でカバーする。 |
| 12. 試料・情報(研究に用いられる情報に係る資料を含む。)の保管及び廃棄の方法<br>指針第7(1)⑩ | (1)試料の保管等<br>情報等については当該論文等の発表後(もしくは研究終了報告後)より 10 年、試料については 5 年保管する。資料(文書、数値データ、画像など)の保存期間は、当該論文等の発表後 10 年間とする。電子化データについては、メタデータの整理・管理と適切なバックアップの作成により再利用可能な形で保存する。<br>なお、紙媒体の資料等についても少なくとも 10 年保存する。保管スペースの確保が困難な場合には裁断して破棄する。<br><br>(2)試料の廃棄<br>廃棄は紙媒体の試料は学内で裁断し破棄する。電子データは保存期間終了後に速やかに削除する。<br><br>(3)情報・データ等の項目<br>年齢、性別、身長、体重、関節角度、歩幅<br><br>(4)情報・データ等の入手方法<br>同意を得られた対象者に対し障害物回避動作の動作計測を行う。<br><br>(5)情報・データ等の収集方法 [調査票の添付　□あり ■なし]<br><br>・音声・画像等の記録<br>■①なし<br>□②あり<br>具体的な記録の内容:<br><br>(6)情報・データの保管(原則:保管)<br><br>ア. 研究期間中の保管場所 :小田原保健医療学部理学療法学科 601 研究室<br>保管方法 :パスワードをかけた電子データ、紙媒体の試料は鍵付きのロッカーで保管<br>イ. 研究終了後の保管場所 :小田原保健医療学部理学療法学科 601 研究室<br>保管方法 :パスワードをかけた電子データ、紙媒体の試料は鍵付きのロッカーで保管<br>ウ. 保管期間　西暦 2033 年 3 月 31 日迄<br><br>廃棄が必要な場合はその理由:<br><br>(7)将来別の研究にデータ等を使用する可能性または別の機関に提供する可能性の有無 : |

| | |
|---|---|
| | ■①ない<br>□②ある<br>具体的に:<br><br>(8)情報・データ等の破棄の方法<br>廃棄は紙媒体の試料は学内で裁断し破棄する。電子データは保存期間終了後に速やかに削除する。 |
| 13．研究機関の長への報告内容及び方法<br>指針第7(1)⑪ | 研究責任者は以下について文書により研究機関の長に報告する。<br>① 研究の進捗状況及び研究の実施に伴う有害事象の発生状況<br>② 研究の倫理的妥当性若しくは科学的合理性を損なう事実若しくは情報又は損なうおそれのある情報であって研究の継続に影響を与えると考えられるものを得た場合<br>③ 研究の実施の適正性若しくは研究結果の信頼を損なう事実若しくは情報又は損なうおそれのある情報を得た場合<br>④ 研究が終了(停止・中止)した場合<br>⑤ 重篤な有害事象が発生した場合<br>研究に関連する情報の漏えい等、研究対象者等の人権を尊重する観点又は研究の実施上の観点から重大な懸念が生じた場合<br>研究実施状況報告書は研究が継続している限り毎年提出する。 |
| 14．研究の資金源等<br>指針第7(1)⑫ | ■学内<br>■①学内研究費<br>□②その他( )<br>■学外<br>■③科学研究費助成事業(学術研究助成基金助成金／科学研究費補助金)<br>□④厚生労働科学研究費補助金<br>□⑤その他の公的研究費( )<br>□⑥企業等からの研究費(受託・共同研究)<br>□⑦企業等からの寄付金<br>□研究費は必要としない<br>□その他( ) |
| 15．研究機関の研究に係る利益相反及び個人の収益等、研究者等の研究に係る利益相反に関する状況<br><br>指針第7(1)⑫ | (1)利益相反<br><br>■ 本研究に係る利益相反はない。<br>□ 本研究には以下の利益相反が存在する。<br>利益相反の内容および対処法(具体的に記載)<br><br>(2)企業等との経済的利益関係<br>■①企業等との関わりはない<br>□②受託研究として実施<br>委託元機関名 :<br>□③共同研究として実施<br>共同研究先機関名:<br>□④企業等に業務委託を行う<br>委託先機関名 :<br>委託内容 :<br>委託先の監督方法:<br>□⑤その他( ) |

| | |
|---|---|
| | ア .上記に記載した企業等との間の経済的利益関係の有無<br>□①ない<br>□②ある(以下を記入)<br>経済的利益関係について:<br>イ .研究結果・対象者保護に影響を及ぼす可能性の有無<br>□①ない<br>□②ある(以下(3)を記入)<br><br>(3)想定される影響の内容:<br>特になし |
| 16. 研究に関する情報公開の方法<br>指針第 7(1)⑬ | (1)公的データベースへの登録<br>□ jRCT(Japan Registry of Clinical Trials)<br>■ 大学病院医療情報ネットワーク研究センター 臨床試験登録システム(UMIN-CTR)<br>(試験ID: UMIN000050796 )<br>□日本医師会治験促進センター臨床試験登録システム(JMA CCT)<br>(試験ID: )<br>□一般財団法人日本医薬情報センターJAPIC 医薬品データベース<br>(試験ID: )<br>□ その他( )<br>□登録なし<br>登録しない理由を記載( )<br><br>(2)研究結果の発表・公開<br>研究終了後の論文投稿を行い結果の公表を行う。 |
| 17. 研究対象者等及びその関係者からの相談等への対応<br>指針第7(1)⑮ | 相談窓口:責任者 須藤大輔<br>所属:国際医療福祉大学 小田原保健医療学部 理学療法学科<br>職名:助教<br>連絡先:d.sudo@iuhw.ac.jp(平日 9:00～1700) |
| 18. 代諾者等からインフォーム | ■ 該当しない<br>代諾者等からインフォームド・コンセントを受ける場合の手続き(代諾者等の選定方針並びに説明及び同意に関する事項を含む。) |

| | |
|---|---|
| ド・コンセントを受ける場合<br>指針第7(1)⑯ | (1)代諾者による同意が必要な研究対象者とその理由<br>該当なし<br><br>(2)代諾者等の選定方針<br>該当なし<br><br>(3)代諾者等への説明事項<br>該当なし<br><br>(4)当該者を研究対象者とすることが必要な理由<br>該当なし<br><br>以下の書式を用いてもよい。<br>□代諾者から文書によるインフォームド・コンセントを得る<br>対象者の属性:<br>代諾者:<br>□親権者または未成年後見人<br>□その他(具体的に: ) |
| 19. インフォームド・アセントを得る場合<br>指針第7(1)⑰ | ■ 該当しない<br>インフォームド・アセントを得る場合の手続(説明に関する事項を含む。) |
| 20. 研究対象者に緊急かつ明白な生命の危機が生じている状況における研究を実施しようとする場合<br>指針第8の8 | ■ 該当しない<br>要件の全てを満たしていることについて判断する方法 |
| 21. 研究対象者等に経済的負担又は謝礼がある場合<br>指針第7(1)⑲ | □ 該当しない<br>その旨及びその内容<br>(1)対象者への謝礼<br>□①謝礼、交通費等は支払わない<br>■②交通費等の実費を支払う<br>■③謝礼を支払う(具体的に:謝礼 10000 円を高齢者の被験者に支払う )<br>(2)対象者の経済的負担<br>■①経済的負担はない<br>□②経済的負担がある(具体的に: ) |
| 22. 通常の診療を超える医療行為を伴う研究の場合<br>指針第7(1)㉒ | ■ 該当しない |

研究対象者への研究実施後における医療の提供に関する対応

23. 研究の実施により得られた結果等の説明に係る手続き、および研究対象者の健康、子孫に受け継がれ得る遺伝的特徴等に関する重要な知見が得られる可能性がある場合の取り扱い
指針第10

以下の内容を了承しました。
■ はい
□ いいえ

私、研究責任者は、実施しようとする研究及び当該研究により得られる結果等の特性を踏まえ、当該研究により得られる結果等の研究対象者への説明方針を定め、研究計画書に記載いたします。当該方針を定める際には、次に掲げる事項について考慮しております。
ア 当該結果等が研究対象者の健康状態等を評価するための情報として、その精度や確実性が十分であるか
イ 当該結果等が研究対象者の健康等にとって重要な事実であるか
ウ 当該結果等の説明が研究業務の適正な実施に著しい支障を及ぼす可能性があるか

偶発的所見もしくは研究対象者の子孫に受け継がれ得る遺伝的特徴等に関する重要な知見が得られる可能性
■ 該当しない

(1)研究対象者に係る研究結果(偶発的所見を含む)の取り扱い
ア. 対象者の健康等に関する重要な知見(偶発的所見を含む)が得られる可能性
□①ない
□②ある

具体的に:
イ. 対象者への研究結果の開示の方針 (ア②の場合)
□①研究結果の開示を行わない
(理由:　　　　　　　　　　　　　　　　)
□②研究結果の開示を行う
□すべて開示する
□一部についてのみ開示する
(理由:　　　　　　　　　　　　　　　　)
(開示の求めを受け付ける方法・開示を行う条件:　　　　　　　　　　　)
(開示の方法:　　　　　　　　　　　　　　　　　　　　　　　　　　　)

| | |
|---|---|
| 24．研究に関する業務の一部を委託する場合<br>指針第7(1)㉓ | ■ 該当しない |
| | 当該業務内容及び委託先の監督方法 |
| | |
| 25．研究対象者から取得された試料・情報について、研究対象者等から同意を受ける時点では特定されない将来の研究のために用いられる可能性がある場合<br>指針第7(1)㉔ | ■ 該当しない |
| | その旨と同意を受ける時点において想定される内容 |
| | |
| 26．モニタリング及び監査を実施する場合<br>指針第7(1)㉕ | ■ 該当しない |
| | その実施体制及び実施手順 |
| | モニタリング実施体制<br>従事者　氏名：<br>所属：<br>資格：<br>実施方法・実施手順：<br><br>監査実施体制<br>従事者　氏名：<br>所属：<br>資格：<br>実施方法・実施手順： |


参考資料・文献リスト：

1) Renato Moraes , et al : Strategies and determinants for selection of alternate foot placement during human locomotion: influence of spatial and temporal constraints. Exp Brain Res. 2004.159(1):1-13.

2) Aram Kim, et al : The quality of visual information about the lower extremities influences visuomotor coordination during virtual obstacle negotiation. J Neurophysiology.2018.120:839-847.

3) Ashwini Kulkarni, et al : Step length synergy is weaker in older adults during obstacle crossing. J Biomech.2021.30:110311.

4) 須藤大輔・他：高齢者における身体機能と身体能力認識が隙間通過時の運動戦略に及ぼす影響．理学療法学．2021．48(1)9-18.


以上
