# Supplementary material for: Effect of vision training on visual function in older adults: A pilot randomized trial
Source: PLoS One. 2026 Aug 24;21(8):e0356206. doi: 10.1371/journal.pone.0356206 (PMC13502553; doi:10.1371/journal.pone.0356206)
Supplement: Supplementary file 4 [file pone.0356206.s004.pdf]

Full English Translation of the Original Study Protocol

Original document: Research Protocol, Version 0.2, May 11, 2023

Study title: Development of a vision training method for acquiring safe obstacle-crossing movements during walking

Translator note: This document is an English translation prepared from the Japanese original research protocol for submission as a full-length protocol translation. The original Japanese protocol format included institutional ethics-review checkboxes and form-specific fields; these have been translated and preserved as faithfully as possible in a readable English format.

Document Information

Item	English translation
Ethics guideline form	Ethical Guidelines for Life Science and Medical Research Involving Human Subjects
Institution	International University of Health and Welfare
Document type	Research Protocol
Version	Version 0.2
Date	May 11, 2023
Principal investigator	Daisuke Sudo
Affiliation	Department of Physical Therapy, School of Health Sciences at Odawara, International University of Health and Welfare
Position	Assistant Professor
Institutional approval statement before submission	The principal investigator reported this protocol to the department head before submission and obtained acknowledgement. Checked: Yes.

1. Title of the Research

Development of a vision training method for acquiring safe obstacle-crossing movements during walking.

2. Research Implementation System

This section corresponds to the research implementation structure, including the names of research institutions and researchers.

2.1 Researchers at the research institution or collaborating research institutions

Researcher	Research institution	Affiliation and position	Qualifications, role, and responsibilities
Daisuke Sudo	International University of Health and Welfare	Department of Physical Therapy, School of Health Sciences at Odawara; Assistant Professor	Principal investigator; data measurement; data analysis
Yusuke Maeda	International University of Health and Welfare	Department of Physical Therapy, School of Health Sciences at Odawara; Associate Professor	Data measurement; data analysis; research supervision
Masato Hosokawa	International University of Health and Welfare	Department of Physical Therapy, School of Health Sciences at Odawara; Assistant Professor	Data measurement; data analysis

2.2 Research cooperation institutions and other non-reviewed institutions

Not applicable.

2.3 Outsourcing of research-related work

Not applicable.

3. Research Period

From the date of approval until March 31, 2027.

4. Purpose and Significance of the Research

4.1 Purpose and significance

Insufficient effects have been obtained even when motor function is improved for the purpose of fall prevention. For older adults to safely avoid obstacles, it is important to perceive the relationship between the body and the environment based on visual information and to adjust posture and gait. However, evidence for this approach as fall prevention has not yet been established.

The applicant has conducted research on passing through gaps as a task involving perception of the self and the environment, and found that older adults at high risk of falling show a rapid reduction in step length and pelvic rotation immediately before passing through a gap. The applicant considered that dependence on central vision due to decreased visual function may be involved. Therefore, the present study aims to clarify the effects of vision training for acquiring safe obstacle-avoidance movements.

By incorporating an approach to gaze that has been used for athletes into interventions for older adults, the coordination between vision and body movement may improve, rapid fluctuations in step length may be prevented, and fall prevention may be supported.

4.2 Expected medical contribution and significance

For obstacle-crossing movements, which are associated with many falls, the applicant considers that the way visual information is acquired may be a causal factor. This study will develop an approach to gaze, which is important when adjusting the relationship between the body and the environment, and will clarify the effect of preventive training. The study is expected to contribute to community-based fall-prevention approaches.

5. Research Methods

5.1 Overview of the research methods

Forty older adults will be divided into two groups by random allocation using a random number table. One group will receive training focused mainly on lower-limb and trunk muscle strengthening, and the other group will receive lower-limb and trunk muscle strengthening plus vision training.

Before and after training, the two groups will be compared with respect to changes in hip, knee, and ankle joint angles; hip, knee, and ankle joint moments; duration of gaze fixation on the obstacle; step length; the distance between the obstacle and the toe at takeoff; and visual function during obstacle-crossing movements.

Visual function will be evaluated using V-training, which is recommended by the Japan Sports Vision Association. The evaluation will include visual acuity, eye-hand/body coordination, instantaneous visual memory, peripheral visual field detection ability, spatial cognitive ability, and eye movements.

After the initial assessment, each training program will be performed three times per week for 8 weeks. Then, three-dimensional motion analysis of gap-passing movements, gaze measurement, and visual function assessment will be performed again.

For comparison, gap-passing movement measurement and gaze measurement will also be performed in 20 younger adults.

The vision training method will follow recommendations of the Japan Sports Vision Association and will involve exercises that move the eyes, with the aim of improving peripheral detection ability. To prevent falls due to dizziness or unsteadiness, vision training will be performed in a seated position three times per week for 8 weeks.

Paired t-tests will be conducted to compare outcomes before and after training. Dropout cases will be excluded from the analysis dataset.

5.2 Research design

This is an interventional study conducted at International University of Health and Welfare alone, with the purpose of verifying the effect of vision training using three-dimensional motion analysis and visual function assessment equipment.

5.3 Pharmaceuticals, medical devices, treatment methods, and observation methods used in this study

Not applicable.

5.4 Outcomes and assessment methods

Primary outcomes: step length and duration of gaze fixation on the obstacle.

Secondary outcomes: hip joint angle, knee joint angle, and ankle joint angle.

5.5 Statistical analysis methods

Forty older adults will be divided into two groups by random allocation using a random number table. One group will receive training focused mainly on lower-limb and trunk muscle strengthening, and the other group will receive lower-limb and trunk muscle strengthening plus vision training. Before and after training, changes will be compared in hip, knee, and ankle joint angles; hip, knee, and ankle joint moments; duration of gaze fixation on the obstacle; step length; the obstacle-to-toe distance at takeoff; and visual function during obstacle crossing.

After the 8-week vision training period performed three times per week, gap-passing movement will be measured again using three-dimensional motion analysis, gaze measurement, and visual function assessment. For comparison, gap-passing movement measurement and gaze measurement will also be performed in 20 younger adults. The statistical method will be paired t-tests comparing pre-training and post-training values. Dropout cases will be excluded from the analysis dataset.

5.6 Planned number of research participants

- Younger adults: 20 participants.
- Older adults: 40 participants.

5.7 Rationale for the number of participants

The target number of older adult participants was set at 40 based on sample size calculation using an effect size of 0.5, alpha error of 0.05, and statistical power of 0.8, together with trends observed in previous studies.

References cited in the protocol for this rationale: Efficacy of a Digital Sports Vision Training Program for Improving Visual Abilities in Collegiate Baseball and Softball Athletes, 2021; Testing the efficacy of vision training for presbyopia: alternating-distance training does not facilitate vision improvement compared to fixed-distance training, 2022.

5.8 Planned participation period for research participants

Initial measurement day, training period of three sessions per week for 8 weeks, and final measurement day.

5.9 Samples/information used in the research and collection schedule

Time point	Data and assessments
Initial measurement day	Task movement measurement: obstacle-crossing movement measured using a three-dimensional motion analysis system. Physical function assessment: fall history, single-leg standing time, and Timed Up and Go test. Visual function assessment: eye-hand/body coordination, instantaneous visual memory, peripheral visual field detection ability, spatial cognitive ability, and eye movements.

Training period	Training conducted three times per week for 8 weeks.
Final measurement day	Task movement measurement: obstacle-crossing movement measured using a three-dimensional motion analysis system. Physical function assessment: fall history, single-leg standing time, and Timed Up and Go test. Visual function assessment: eye-hand/body coordination, instantaneous visual memory, peripheral visual field detection ability, spatial cognitive ability, and eye movements.

5.10 Changes to the research protocol and informed consent documents

If the research protocol, informed consent documents, or related materials are changed or revised, implementation will occur only after prior approval has been obtained from the ethics review committee.

5.11 Criteria for discontinuation of research and response to participants

If the principal investigator or a co-investigator determines that continuation of the research is not possible for an individual participant for any of the following reasons, the participant will be informed.

1. Withdrawal of consent by the research participant.
2. Complaint of poor physical condition by the participant.
3. Discontinuation of the overall study according to the criteria described below.
4. Other reasons for which the principal investigator judges that discontinuation of the research is appropriate.

Data from participants whose participation is discontinued will be promptly deleted, and data obtained up to the time of withdrawal will not be used.

5.12 Response to research participants after completion of the research

If participants make inquiries after completion of the research, information will be promptly disclosed, and responses and explanations will be provided.

5.13 Provision of samples or information to other institutions

Not applicable.

6. Policy for Selecting Research Participants

6.1 Overview of participants

- Planned sample size: 60 participants, consisting of 20 younger adults and 40 older adults.
- Sex: all sexes.
- Age range: 20 to 75 years.
- Minors included: No.
- Undergraduate or graduate students of this university or another university included: Yes.
- Adults with insufficient capacity to consent included: No.

Reason why participation of university students is necessary and required considerations: Because the measurement equipment is installed at the university and it is difficult to conduct measurements outside the university, students in the Department of Physical Therapy, School of Health Sciences at Odawara, will be asked to participate as research subjects.

6.2 Participant selection policy

Inclusion criteria:

1. Individuals who, on the measurement day, have no motor paralysis, joint range-of-motion limitations, pain, or other conditions that would interfere with walking.
2. Individuals without cognitive decline who can understand the assessment and measurement procedures.
3. Individuals who can walk independently.

Exclusion criteria:

1. Individuals with visual impairment that interferes with daily life.
2. Individuals who report pain during walking.
3. Individuals who use walking aids.

6.3 Recruitment process

Younger adults will be recruited from students in the Department of Physical Therapy, School of Health Sciences at Odawara, and measurements will be conducted for those who wish to participate.

Older adults will be recruited from a voluntary fall-prevention class with which the applicant is involved.

6.4 Conditions for excluding participants after study start and handling of samples/information

- Withdrawal of consent after study registration (after random allocation).
- Discovery after study registration (after random allocation) of deviation from the inclusion or exclusion criteria.
- Discovery after study registration (after random allocation) of a protocol deviation.

If consent is withdrawn, all samples and information for that case will be destroyed.

6.5 Considerations when undergraduate or graduate students are included

- The recruitment information clearly states that participation or nonparticipation in the research will not affect grades or credit acquisition in courses taught by the researcher.
- Students belonging to the same laboratory as the applicant are not included.
- The explanatory document states that refusal to participate will not affect grades or credit acquisition.
- The consent form will be submitted on a later date after sufficient explanation of the research has been provided.
- Other considerations: none specified.

7. Scientific Rationale for the Research

The study will analyze factors related to older adults tripping over obstacles by using a three-dimensional motion analysis system and visual function assessment, and will verify whether body movement and visual function change after vision training. The planned research period is 5 years.

8. Procedures for Obtaining Informed Consent

8.1 Method of consent procedure

Explanation and consent will be obtained using written documents. When undergraduate students are included, participants will be informed at the time of consent that nonparticipation will not affect grades in any way. Written and oral explanations will be provided before measurement begins. Older adults will also be provided with written explanations and written consent will be obtained. When obtaining consent from older adults, participants will be informed that nonparticipation will not affect their participation in fall-prevention class activities in any way.

8.2 Specific method for obtaining consent

- Method of explanation: oral explanation accompanied by a written document.

- Person providing explanation: principal investigator.

8.3 Withdrawal of consent

If a participant wishes to withdraw consent, participation will be promptly discontinued and collected data will be destroyed. There is no provision of samples or data to other institutions. Even after agreeing to the implementation or continuation of the research, participants may withdraw consent at any time.

9. Handling of Personal Information

This research handles personal information: Yes.

9.1 Types of personal information collected

The study will collect names, dates of birth, and other descriptions or records that can identify specific individuals. Specific contents: name and age. Purpose of use: collection of basic information.

Information represented by voice, movement, or other methods that can identify a specific individual: not selected in the original form. Personal identification codes: not selected. Information that can identify a deceased individual: not selected. Sensitive personal information: not selected.

9.2 Creation of pseudonymized or anonymized information

Pseudonymized information will be created. Person responsible: Daisuke Sudo. Affiliation: Department of Physical Therapy, School of Health Sciences at Odawara. Qualification/role: Principal investigator.

Management of the correspondence table: The correspondence table will be saved on external storage media using a computer disconnected from external networks and will be strictly stored under lock. Paper records will also be strictly stored under lock.

All researchers involved in this study will conduct the research in compliance with the Declaration of Helsinki and the Ethical Guidelines for Life Science and Medical Research Involving Human Subjects. When handling samples and information related to the research, numbers unrelated to the participants' personal information will be assigned and managed, with sufficient attention to protecting the privacy of participants. When research results are published, the results will not include information that can identify participants. Samples and information obtained through the research will not be used for purposes other than the research purpose.

Anonymized information will not be created.

10. Burden, Expected Risks and Benefits, Overall Assessment, and Measures to Minimize Burden and Risk

10.1 Burden on research participants

For three-dimensional motion analysis and visual function measurement, procedures will be confirmed in advance and efforts will be made to minimize the duration of participant restraint. Data collection time will be approximately 60 minutes. Actual transportation expenses associated with participant travel will be paid.

10.2 Expected risks

This is an interventional study. During physical function assessments before and after the intervention, there is a risk of falling when measuring single-leg standing time and obstacle-crossing movement.

10.3 Expected benefits

The vision training performed in this study is expected to produce the benefit of changes in body movement depending on the results.

10.4 Overall assessment and measures to minimize burden and risk

During motion measurement and physical function assessments, including single-leg standing and the Timed Up and Go test, multiple staff members will be present as a fall-prevention measure, and assessments will be performed from positions where immediate assistance can be provided if unsteadiness occurs. Participants' physical condition will be carefully checked. If a participant is unwell, the measurement or assessment will be discontinued.

System for preventing occurrence or minimizing severity of harm: Measurements will always be conducted by two physical therapists.

11. Research Involving Invasiveness Other Than Minimal Invasiveness

The original form indicates not applicable for research involving invasiveness other than minimal invasiveness. The following responses were nevertheless described in the form.

11.1 Response when serious adverse events occur

Initial response will be provided by faculty members of the Department of Physical Therapy. If the situation is judged to be difficult to manage, assistance will be requested from the dean of the faculty, who is a physician, and faculty members of the Department of Nursing. If treatment at a medical institution is necessary, emergency services will be requested promptly.

11.2 Compensation for health damage caused by the research

Regarding compensation and liability, if damage occurs, it will be covered by the investigator's personal insurance, namely the physical therapist association liability insurance.

12. Storage and Disposal of Samples and Information

12.1 Storage of samples and information

Information and related materials will be stored for 10 years after publication of the relevant paper or after submission of the research completion report, and samples will be stored for 5 years. Materials, including documents, numerical data, and images, will be stored for 10 years after publication of the relevant paper. Electronic data will be stored in a reusable format through organization and management of metadata and appropriate backup. Paper materials will also be stored for at least 10 years. If securing storage space is difficult, materials will be shredded and discarded.

12.2 Disposal of samples

Paper samples will be shredded and discarded within the university. Electronic data will be promptly deleted after the storage period ends.

12.3 Information/data items

Age, sex, height, body weight, joint angles, and step length.

12.4 Method for obtaining information/data

Obstacle-avoidance motion measurement will be conducted for participants from whom consent has been obtained.

12.5 Method for collecting information/data

Questionnaire attached: No. Audio or image recording: No.

12.6 Storage of information/data

Period	Storage location	Storage method

During the research period	Department of Physical Therapy, School of Health Sciences at Odawara	Password-protected electronic data; paper samples stored in a locked locker.
After completion of the research	Department of Physical Therapy, School of Health Sciences at Odawara	Password-protected electronic data; paper samples stored in a locked locker.

Storage period: until March 31, 2033.

12.7 Possibility of future use or provision to another institution

There is no possibility that data or information will be used for another future study or provided to another institution.

12.8 Method for destroying information/data

Paper samples will be shredded and discarded within the university. Electronic data will be promptly deleted after the storage period ends.

13. Reporting to the Head of the Research Institution

The principal investigator will report the following matters to the head of the research institution in writing:

1. Progress status of the research and the occurrence status of adverse events associated with research implementation.
2. Facts or information that may impair, or have the potential to impair, the ethical validity or scientific rationality of the research and that are considered to affect continuation of the research.
3. Facts or information that may impair, or have the potential to impair, the appropriateness of research implementation or the reliability of research results.
4. Completion, suspension, or discontinuation of the research.
5. Occurrence of a serious adverse event.
6. Any serious concern from the perspective of respecting the human rights of research participants or from the perspective of research implementation, such as leakage of research-related information.

A research implementation status report will be submitted every year as long as the study continues.

14. Funding Sources

Internal funding: university research funds. External funding: Grants-in-Aid for Scientific Research (KAKENHI; Scientific Research Grant Fund / Scientific Research Grant). Other categories such as Health, Labour and Welfare Sciences Research Grants, other public research funds, entrusted or joint research funds from companies, corporate donations, or no funding required were not selected in the original form.

15. Conflicts of Interest

15.1 Conflicts of interest related to the research institution and individual researchers

There are no conflicts of interest related to this research.

15.2 Economic relationships with companies or similar entities

There is no involvement with companies or similar entities. Entrusted research, joint research, outsourcing to companies, and other relationships were not selected in the original form.

15.3 Potential influence on research results or participant protection

None specified.

16. Method for Public Disclosure of Research Information

16.1 Registration in a public database

The study is registered in the University Hospital Medical Information Network Clinical Trials Registry (UMIN-CTR). Trial ID: UMIN000050796. jRCT, JMA CCT, JAPIC, and other registries were not selected in the original form.

16.2 Publication and disclosure of research results

After the research is completed, the results will be published through journal submission.

17. Informed Consent from Legally Authorized Representatives

Not applicable.

17.1 Participants requiring consent by a legally authorized representative and reason

Not applicable.

17.2 Policy for selecting legally authorized representatives

Not applicable.

17.3 Items to be explained to legally authorized representatives

Not applicable.

17.4 Reason why such persons must be included as research participants

Not applicable.

18. Informed Assent

Not applicable. Procedures for obtaining informed assent and explanation-related matters do not apply.

19. Research Conducted in Situations Where Research Participants Face an Urgent and Obvious Threat to Life

Not applicable.

20. Economic Burden or Honorarium for Research Participants

20.1 Honorarium and transportation expenses

Actual expenses such as transportation expenses will be paid. An honorarium will also be paid: 10,000 yen will be paid to older adult participants.

20.2 Economic burden on participants

There is no economic burden on participants.

21. Research Involving Medical Practice Beyond Usual Medical Care

Not applicable.

22. Procedures for Explaining Results Obtained Through the Research and Handling of Potential Important Findings

The principal investigator acknowledged the following. Taking into account the nature of the research and the results obtained, the investigator will establish a policy for explaining results to research participants and will describe the policy in the research protocol. In establishing this policy, the investigator will consider the following points:

- Whether the results are sufficiently accurate and reliable as information for evaluating the participant's health status.
- Whether the results are important facts related to the participant's health or similar matters.
- Whether explaining the results may significantly interfere with appropriate implementation of the research work.

The possibility of obtaining incidental findings or important findings concerning genetic characteristics that may be inherited by descendants of research participants: not applicable.

22.1 Handling of research results related to participants, including incidental findings

The original form did not specify the possibility of obtaining important findings related to participants' health or a policy for disclosure of research results because this item was not applicable.

23. Outsourcing of Research-Related Work

Not applicable.

24. Potential Future Use of Samples or Information for Unspecified Future Research

Not applicable. The original protocol did not indicate future use of samples or information for research that was unspecified at the time consent was obtained.

25. Monitoring and Auditing

Not applicable. Monitoring and auditing systems, personnel, implementation methods, and procedures were not specified because monitoring and auditing were not planned.

Reference Materials and Literature List in the Original Protocol

1. Moraes R, et al. Strategies and determinants for selection of alternate foot placement during human locomotion: influence of spatial and temporal constraints. Experimental Brain Research. 2004;159(1):1-13.
2. Kim A, et al. The quality of visual information about the lower extremities influences visuomotor coordination during virtual obstacle negotiation. Journal of Neurophysiology. 2018;120:839-847.
3. Kulkarni A, et al. Step length synergy is weaker in older adults during obstacle crossing. Journal of Biomechanics. 2021;30:110311.
4. Sudo D, et al. Effects of physical function and perceived physical ability on motor strategies during gap passage in older adults. Rigakuryoho-gaku. 2021;48(1):9-18. [Japanese].
